# Pathological Society of London

**Published:** 1888-03

**Authors:** 


### NOTES FROM FOREIGN MEDICAL SOCIETIES.

### PATHOLOGICAL SOCIETY OF LONDON.

*Etiology of Vesical Growths.*—A series of specimens intended to show that irritation of various kinds played a most important part in the production of vesical growths were demonstrated by Mr. HURRY FENWICK. He pointed out that the very earliest form of villous papilloma was to be found in a small patch of stunted papilloma known as subvilloid or cropped villi, and in most of these specimens there existed definite evidence of irritation. Thus, in a case of Dr. Beaven Rake's the patch was found at that part of the bladder which impinged against a straw, coated with phosphates, four inches and a half long. In Dr. Newman's case the tumor was at the apex of the bladder, and seemed due to the irritation of a large oval stone. Mr. Fenwick then alluded to age and to the position of villous papillomata as corroborative evidence. In carcinoma he believed that the irritation of residual urine was a most important causative factor, and quoted cases in the museums of Stockholm, Glasgow, and London.

Mr. MARMADUKE SHIELD referred to a case of carcinoma of the bladder which had followed at a considerable interval on epithelioma of the penis; amputation of the penis was followed by stricture; the new growth was attributed to the prolonged irritation of instrumentation and of putrid urine. The specimen had been shown to the society two years ago.

Mr. EVE said that in the specimen he showed of a urinary bladder from a case of bilharzia the thickening of the organ was purely inflammatory.

*Dry Caries.*—Four specimens of dry caries were shown by Mr. EVE, who referred to the occurrence of this condition in syphilis and tuberculosis. One specimen, from the museum of the London Hospital, was an example of angular curvature taken from a man, aged 39, in which no abscess ever formed, and no pus was found after death. A second specimen, from the museum of the Royal College of Surgeons, was also an example of angular curvature from the dorso-lumbar region. There was caseous material and necrosis, but no evidence of suppuration. Two other specimens showed extensive destruction of bone without suppuration. In one (presented to the museum of the Royal College of Surgeons by Mr. Jonathan Hutchinson), there was complete destruction of the cancellous tissue of the oscalcis, which was replaced by fibrous tissue; there was no evidence of tubercle. The cases ought not to be confounded with rarifying osteitis occurring in connection with simple inflammatory action, or with tuberculosis.

Mr. MARMADUKE SHIELD said that many of the cases of dry caries of the spine were associated with caseation, as in one of the specimens shown. In such a case, where the caseous matter was inclosed in a distinct sac, he thought it most probable that there had been suppuration, and that the fluid part had been absorbed, leaving a caseous mass. Paraplegia was not infrequent where abscess was not present, or at least apparent, while where suppuration was present paraplegia seldom occurred.

Mr. MORRANT BAKER thought this was an interesting clinical observation which his experience confirmed.

Mr. R. W. PARKER had found from the examination of a large number of reported cases that abscess occurred less frequently the higher up the vertebral column was diseased, which might account for it.

Dr. ANGEL MONEY agreed with Mr. Marmaduke Shield's statement.

Mr. WALSHAM thought there was no doubt about its being correct.

*Case of Anomalous Sacral Appendage.*—An infant under the care of Mr. EDMUND OWEN was shown to the society. On the lower part of the back was a rounded swelling measuring 3¾ by 3½ inches, and projecting about seven-eighths of an inch. The lower limit was just above the fold of the nates; the tumor, which was situated a little to the left of the middle line, presented in the exact centre an umbilication. Half way between the dimple and the lower border, and slightly to the left of the middle line, was a soft appendage about 2 inches long, and having somewhat the appearance of a fat "little finger"; the base of the appendage was constricted. On the left side of the base of the appendage was a second small excrescence a quarter of an inch long. Both hands were badly developed, being smaller than natural; all other parts of the body appeared to be normal. The child was born at full term, and there was no history of deformity in the family. Mr. Owen suggested that the rounded swelling was the result of spina bifida, while the appendage, and perhaps the tumor also, was the result of an imperfect attempt to produce a double monster; the sacral region was by no means an infrequent site for the attachment of a fairly developed or rudimentary fœtus.

Mr. MORRANT BAKER and Mr. J. B. SUTTON both agreed that the case was an example of parasitic fœtus. Mr. Sutton observed that if during development the medullary fold remained cleft, two complete fœtuses were formed from a single ovum ; this was probably the explanation of twins of the same sex in one amniotic sac. From this there was every degree of combination, from Siamese twins to such a case of very rudimentary fœtus as that shown by Mr. Owen.

*Imperforate Urethra.*—Mr. SHATTOCK showed a fœtus of about four months ; the abdomen was greatly distended, and when opened was found to contain a relatively immense cavity which almost filled it ; opening into this large cavity the two ureters could be seen. The kidneys were in a condition of cystic disease. The intestine terminated in a well-formed rectum. The urethra was closed. Mr. Shattock discussed the mode of production of this and other similar deformities in a paper of some length, and involving very technical embryological matters.

OBSTETRICAL SOCIETY OF LONDON.

